# Origins of Dirac cone formation in AB_3_ and A_3_B (A, B = C, Si, and Ge) binary monolayers

**DOI:** 10.1038/s41598-017-10670-x

**Published:** 2017-09-05

**Authors:** Xuming Qin, Yuqin Wu, Yi Liu, Baoqian Chi, Xiaowu Li, Yin Wang, Xinluo Zhao

**Affiliations:** 1 Department of Physics, Materials Genome Institute, and International Centre for Quantum and Molecular Structures, Shanghai University, 99 Shangda Road, Shanghai, 200444 P.R. China; 2 Department of Materials Physics and Chemistry, School of Materials Science and Engineering, and Key Laboratory for Anisotropy and Texture of Materials (Ministry of Education), Northeastern University, No. 3-11 Wenhua Road, Shenyang, 110819 P.R. China

## Abstract

Compared to the pure two-dimensional (2D) graphene and silicene, the binary 2D system silagraphenes, consisting of both C and Si atoms, possess more diverse electronic structures depending on their various chemical stoichiometry and arrangement pattern of binary components. By performing calculations with both density functional theory and a Tight-binding model, we elucidated the formation of Dirac cone (DC) band structures in SiC_3_ and Si_3_C as well as their analogous binary monolayers including SiGe_3_, Si_3_Ge, GeC_3_, and Ge_3_C. A “ring coupling” mechanism, referring to the couplings among the six ring atoms, was proposed to explain the origin of DCs in AB_3_ and A_3_B binary systems, based on which we discussed the methods tuning the SiC_3_ systems into self-doped systems. The first-principles quantum transport calculations by non-equilibrium Green’s function method combined with density functional theory showed that the electron conductance of SiC_3_ and Si_3_C lie between those of graphene and silicene, proportional to the carbon concentrations. Understanding the DC formation mechanism and electronic properties sheds light onto the design principles for novel Fermi Dirac systems used in nanoelectronic devices.

## Introduction

The graphene synthesized in 2004^1^ have aroused enormous theoretical and experimental interests on two dimensional (2D) materials. Besides graphene, some other pure 2D materials were proposed such as graphyne or graphdiyne^2–5^, silicene^6–8^, germanene^8^, phosphorene^9, 10^, and borophene^11, 12^, where graphdiyne^3, 5^, silicene^7^, black phosphorene^9^, and borophene^13^ have been synthesized experimentally. In addition to pure 2D materials, the studies of binary or multivariate 2D materials were carried out gradually. In 2011, it was reported that monolayers were exfoliated from the layered compounds such as MoS_2_ in some common solvents, providing a strategy to synthesize 2D crystals^14^. Inspired by graphene and silicene, binary 2D monolayers consisting of C and Si dubbed silagraphene exhibit rich structures including various chemical stoichiometry and arrangement patterns associated with different electronic properties.

The synthesis of silicon carbide nanotubes^15, 16^ offered a possibility to prepare 2D crystals silagraphenes and several theoretical studies about silagraphene were carried out. Among various silagraphenes, the most commonly studied structure has C/Si = 1:1 ratio with alternative C and Si arrangement dubbed h-SiC in this paper. The first-principles calculations predicted that h-SiC was a semiconductor^17–22^. Chen *et al*. reported that a fully hydrogenated/fluorinated h-SiC heterobilayer possessed a quasi-metallic character and an external electric field opened a direct band gap, implying the potential applications in future nanoelectronics and optoelectronics^23^. Wang *et al*. showed that h-SiC can be used as metal-free catalyst for CO oxidation^24^. Keeping C/Si = 1:1 stoichiometry but varying the arrangement patterns, we demonstrated previously that t1-SiC and t2-SiC featuring C–C and Si–Si pairs were semimetal with Dirac cone (DC) featured band structures^25^. Silagraphenes with other stoichiometry have also been studied recently. The density functional theory (DFT) calculations combined with many-body perturbation formalism revealed that the band gap of silagraphene can be tuned continuously by varying the concentration of Si^26^. SiC_2_ was predicted to possess a metallic planar structure with local minimum featuring planar tetracoordinate Si units^27^. Further global structure search predicted that SiC_2_ prefers to form three buckled structures using the particle swarm optimization method with dispersed C_2_ dimers rather than individual C atoms^28^. Recently the first-principles calculations predicted that SiC_7_ silagraphene is a semiconductor with a direct band gap of 1.13 eV^29^. g-SiC_3_ and g-Si_3_C are predicted to possess DC band structures^30, 31^. Meanwhile, significant band gaps are opened and the band structures are topologically nontrivial after the introduction of spin-orbital coupling^30^.

DC featured band structures commonly lead to unique electronic properties. For example, the charge carrier mobility of graphene which possess DC band structure^32, 33^ can reach up to 10^7^ cm^2^ /(V s)^34^. Only a few 2D materials possess DCs. The pure 2D DC materials include graphyne^4, 35^, square graphynes^36^, silicene^6, 8^, germanene^8^, and borophene^37^. The binary 2D DC systems include g-SiC_3_, g-Si_3_C^30, 31^, t1-SiC, t2-SiC^25^, and silagraphye^38, 39^. The modified 2D DC systems include 6(H_2_), 14, 18 graphyne, 6_BN_, 6, 12 graphyne^40^, janugraphene, chlorographene^41^, and hydrogenated and halogenated blue phosphorene^42^. The organic 2D DC systems include Mn_2_C_18_H_12_^43^ and Ni_2_C_24_S_6_H_12_^44^.

Despite many reports on 2D DC systems, fewer studies contribute to the origin of DC formation. Using a two bands model, Wang *et al*. summarized that the conditions of DC formation include specific symmetries, proper parameters, and a suitable Fermi level where there are only DC points and no other bands^45^. To understand the origin of DC of graphyne, it was clarified that the acetylenic linkages between vertexes atoms could be reduced to effective hopping terms whose combination decides the existence of DCs^46, 47^. More recently, by performing calculations using both DFT and a tight binding (TB) model. We proposed “pair coupling”^25^ and “triple coupling”^39^ mechanisms to elucidate the origin of DC formation of t1-SiC and α-graphyne, showing different processes of DC formations.

In this work, by performing DF and TB calculations, we analyzed the formation process of band structures of g-SiC_3_ and g-Si_3_C and elucidated the origin of DC formation by proposing a “ring coupling” mechanism referring to the couplings among the six same atoms forming a ring. On the basis of this mechanism, the conditions of the systems being self-doped were also discussed. Furthermore, we verified the “ring coupling” mechanism by studying analogous binary monolayers consisting of Ge and C as well as Ge and Si, showing DC featured band structures consistent with the results of Zhao *et al*.^30^. Finally, we calculated the electron transport properties of g-SiC_3_ and g-Si_3_C using non-equilibrium Green’s function method combined with density functional theory (NEGF-DFT), showing that the studied silagraphene exhibit electron conductance between silicene and graphene.

## Results and Discussion

### Atomic structures and stability of g-SiC_3_ and g-Si_3_C

**Figure 1:**
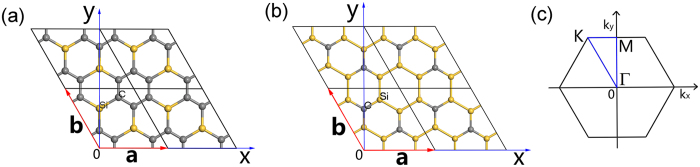
Atomic structures of (**a**) g-SiC_3_, (**b**) g-Si_3_C. (**c**) Brillouin zone models of all structures in this work.

By geometry optimization using DFT calculation, the atomic structures of g-SiC_3_ and g-Si_3_C (shown in Fig. 1) are acquired. They have planar forms with P6/MMM symmetry. And they are both graphene-like but consisting of two elements, one of which forms 6-membered rings. The corresponding structure parameters and formation energies are listed in Table 1. For comparison, the results of graphene and silicene from our previous work^39^ are also listed in Table 1.

**Table 1:** Bond lengths *l* (Å), lattice parameters *a* (Å), and formation energies per atom ${E}_{f}$, ${E}_{f^{\prime} }$ (eV), and the electron (hole) group velocities near Fermi surface *v* of g-SiC_3_, g-Si_3_C, graphene, and silicene. ^a^From ref. 39.

A_x_B_y_	*l*_A-A_/*l*_B-B_	*d*_A-B_	*a*	${{\boldsymbol{E}}}_{{\boldsymbol{f}}}$	${{\boldsymbol{E}}}_{{\boldsymbol{f}}^{\prime} }$	***v*** (10^6^ m/s)
g-SiC_3_	1.44	1.81	5.63	7.84	−0.28	0.6
g-Si_3_C	2.25	1.81	7.04	5.73	−0.16	0.5
Graphene	1.42^a^		2.47^a^	9.23^a^		0.8
Silicene	2.28^a^		3.87^a^	4.77^a^		0.5

To analyze the stability of the structures, we calculated two types of formation energy^39^. The first formation energy ${E}_{f}$ is defined as:

1 $${E}_{f}=(\sum _{i}{E}_{i}-{E}_{t})/n$$

where ${E}_{i}$ is the isolated atom energy for the *i*-th atom, ${E}_{t}$ is the total energy per cell, *n* is the total number of atoms per cell. The second formation energy is defined as:

2 $${E}_{f^{\prime} }=(\sum _{i}{n}_{ele-i}{E}_{ele-i}-{E}_{t})/n$$

where ${n}_{ele-i}$ is the number of the atoms of the *i*-th element in a cell, ${E}_{ele-i}$ is the energy per atom of the graphene-like structure only consisting of the *i*-th element (for example, when the *i*-th element is Si, ${E}_{ele-i}$ means the energy per atom of silicene), *n* is the total number of atoms per cell.

With higher C/Si proportion the formation energy ${E}_{f}$ of g-SiC_3_ is higher than g-Si_3_C, consistent with the fact that graphene is more stable than silicene. Compared with graphene and silicene, the C-C and Si-Si bond-lengths of g-SiC_3_ and g-Si_3_C change about 0.01 and 0.02 Å, respectively, and their formation energies ${E}_{f^{\prime} }$ are negative, indicating that the energy of g-SiC_3_ or g-Si_3_C is higher than the ideal mixture of graphene and silicene with the same C/Si proportions as g-SiC_3_ or g-Si_3_C.

We discussed the possibility of atomic segregation into the graphene and silicene nanoribbons with Si-C interfaces in section S2 of Supplementary Information.

To verify the structure stability, we carried out quantum molecular dynamics (MD) calculations at a canonical ensemble (NVT ensemble) at 600 K. The MD trajectories indicate that the atomic structures of g-SiC_3_ and g-Si_3_C do not change significantly after 2.5 ps (See Figure S1 in Supplementary Information). Previous phonon calculations of g-SiC_3_ and g-Si_3_C by Zhao *et al*. did not find modes with imaginary frequencies^30^. Ding *et al*. also verified the stability of g-SiC_3_ by density-functional-based tight binding molecular dynamics simulations and phonon calculations^31^.

### Band structures of g-SiC_3_ and g-Si_3_C

In this work, the Brillouin zones of all the structures possess same models with hexagon shown in Fig. 1(c).

### Band structure of g-SiC_3_

**Figure 2:**
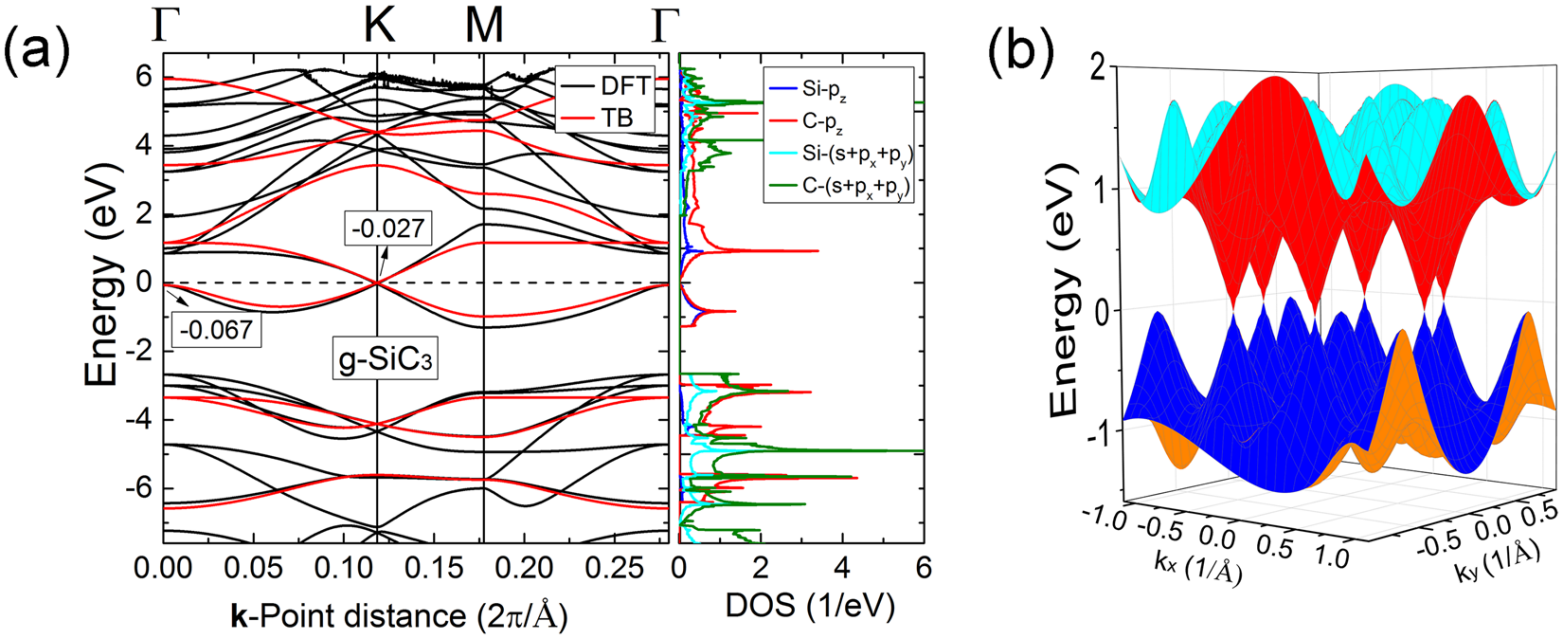
(**a**) Band structure (left) and DOS (right) of g-SiC_3_. For the band structures, the black line is the DFT results and the red line is the results calculated by TB. (**b**) 3D band structure of g-SiC_3_ calculated by DFT.

The band structure of g-SiC_3_ possesses DCs calculated by DFT as shown in Fig. 2(a) and (b). The electron/hole group velocity of g-SiC_3_ near Fermi surface is listed in Table 1. For comparison, the electron/hole group velocities of graphene and silicene were also calculated and listed in Table 1. These values are the group velocities averaged over electrons and holes as well as different directions. The averaged group velocity of g-SiC_3_ is lower than that of graphene but higher than that of silicene.

**Figure 3:**
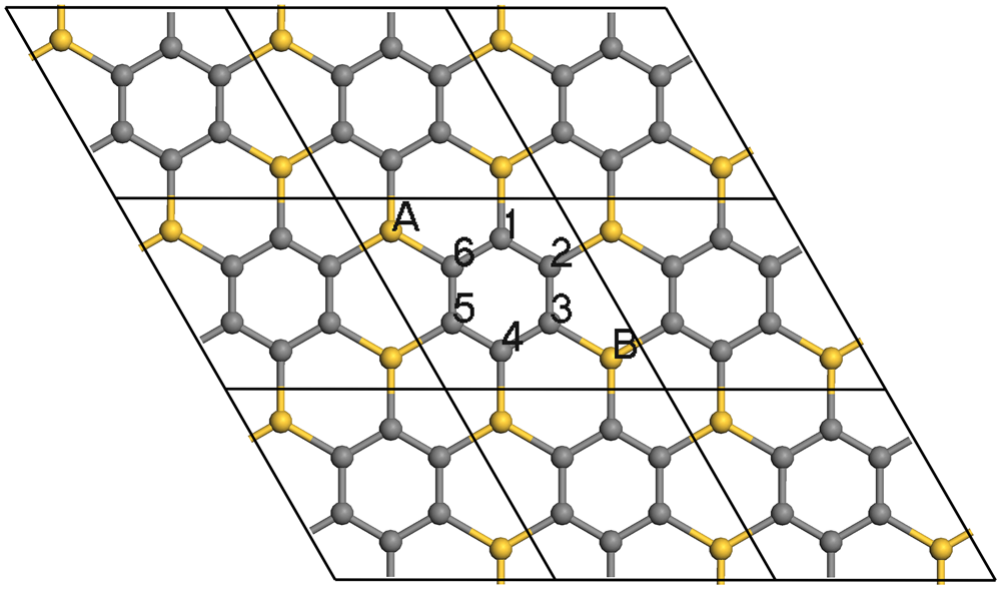
Atomic structure of g-SiC_3_ used for TB analysis.

From the density of states (DOS) of g-SiC_3_ (Fig. 2(a)), the bands near Fermi energy mainly attribute to the p_z_ orbitals of Si and C. So we constructed a TB model to reproduce the band structure by only considering the p_z_ orbitals. For the sake of convenience, we translated properly the lattice of g-SiC_3_ as Fig. 3, and labeled the vertex atoms A and B as well as the ring atoms 1–6. The TB Hamiltonian can be written as^30, 39^:

3 $$\hat{H}=\sum _{i}{E}_{i}{\hat{a}}_{i}^{+}{\hat{a}}_{i}+\sum _{\langle m,n\rangle }(-{t}_{mn}{\hat{a}}_{m}^{+}{\hat{a}}_{n}+{\rm{H}}{\rm{.c}}{\rm{.}})$$

where ${E}_{i}$ is the onsite energy of the *i*-th atom, $-{t}_{mn}$ is the hopping energy between the *n*-th and *m*-th atom (only considering the nearest-neighboring atoms for simplicity), ${\hat{a}}_{i}^{+}$ and ${\hat{a}}_{i}$ are creation and annihilation operators, respectively. The TB parameters are determined by fitting against DFT results^25^. The onsite energies of C and Si are *E*_*C*_ = −1.090 eV and *E*_*Si*_ = 2.459 eV, respectively. The hopping energies of C-C and C-Si are *t*_*C-C*_ = 2.258 eV and *t*_*C-Si*_ = 1.715 eV, respectively. The agreement between the TB and DFT results verifies the rationality of the TB model (See Fig. 2(a)).

To understand the origin of DC featured band structure of g-SiC_3_, we make the analysis based on a TB model as follows. For simplicity, we rewrite *t*_*C-Si*_ as *t*, and *t*_*C-C*_ as *t*_*C*_.

The couplings among the six C ring atoms are strong due to the same onsite energies. So we first only consider the couplings among the six neighbouring C atoms without considering the couplings between the ring atoms and vertex atoms. The corresponding Hamiltonian can be written as:

4 $${\hat{H}}_{C}=\sum _{l=1}^{6}{E}_{C}{\hat{a}}_{l}^{+}{\hat{a}}_{l}-\sum _{m=1}^{6}({t}_{C}{\hat{a}}_{m}^{+}{\hat{a}}_{m+1}+{t}_{C}{\hat{a}}_{m+1}^{+}{\hat{a}}_{m})$$

where *l* or *m* are the atom labels for the six C ring atoms shown in Fig. 3, ${\hat{a}}_{7}$ and ${\hat{a}}_{7}^{+}$ mean ${\hat{a}}_{1}$ and ${\hat{a}}_{1}^{+}$, respectively. The eigenfunctions are:

5 $${|{\phi }_{C-j}\rangle }_{n}=\frac{1}{\sqrt{6}}\sum _{l=1}^{6}{e}^{i\frac{\pi }{3}j\cdot l}{|{\varphi }_{C-l}\rangle }_{n}(j=1,2,\cdots ,6)$$

where ${|{\varphi }_{C-l}\rangle }_{n}$ is the wave function of *l*-th C atom in the *n*-th cell. The conclusion that ${|{\phi }_{C-j}\rangle }_{n}$ is the eigenfunctions of ${\hat{H}}_{C}$ can be verified as:

6 $${\hat{H}}_{C}{|{\phi }_{C-j}\rangle }_{n}=({E}_{C}-{e}^{i\frac{\pi }{3}j}{t}_{C}-{e}^{-i\frac{\pi }{3}j}{t}_{C}){|{\phi }_{C-j}\rangle }_{n}(j=1,2,\cdots ,6)$$

The eigenvalues of $|{\phi }_{C-3}\rangle$ and $|{\phi }_{C-6}\rangle$ are ${E}_{C}+2{t}_{C}$ and ${E}_{C}-2{t}_{C}$, respectively. $|{\phi }_{C-2}\rangle$ and $|{\phi }_{C-4}\rangle$ are degenerated at ${E}_{C}+{t}_{C}$; $|{\phi }_{C-1}\rangle$ and $|{\phi }_{C-5}\rangle$ are degenerated at ${E}_{C}-{t}_{C}$. There are four eigenvalues in total.

Based on the wave functions ${|{\phi }_{C-j}\rangle }_{n}$ and the wave functions of Si atoms, we define the Bloch basis sets:

7 $${|j\rangle }_{{\bf{k}}}=\frac{1}{\sqrt{N}}\sum _{n}{e}^{i{\bf{k}}\cdot {{\bf{R}}}_{n}}{|{\phi }_{C-j}\rangle }_{n}j=1,2,\ldots ,6$$

8 $${|A\rangle }_{{\bf{k}}}=\frac{1}{\sqrt{N}}\sum _{n}{e}^{i{\bf{k}}\cdot {{\bf{R}}}_{n}}{|{\phi }_{Si-A}\rangle }_{n}$$

9 $${|B\rangle }_{{\bf{k}}}=\frac{1}{\sqrt{N}}\sum _{n}{e}^{i{\bf{k}}\cdot {{\bf{R}}}_{n}}{|{\phi }_{Si-B}\rangle }_{n}$$

where ${|{\phi }_{Si-A}\rangle }_{n}$ and ${|{\phi }_{Si-B}\rangle }_{n}$ are the wavefunctions of Si atoms labeled A and B shown in Fig. 3. The eigenfunctions of this system are the linear superposition of these eight functions. With these eight functions as basis vectors in the order of

10 $${|A\rangle }_{{\bf{k}}},{|1\rangle }_{{\bf{k}}},{|2\rangle }_{{\bf{k}}},\,\cdots ,{|6\rangle }_{{\bf{k}}},{|B\rangle }_{{\bf{k}}}$$

The matrix $H({\bf{k}})$ of Hamilton operator $\hat{H}$ can be written as

11 $$H({\bf{k}})=(\begin{array}{lllll}{H}_{AA} & {H}_{A1} & {H}_{A2} & \cdots  & {H}_{AB}\\ {H}_{1A} & {H}_{11} & {H}_{12} & \cdots  & {H}_{1B}\\ {H}_{2A} & {H}_{21} & {H}_{22} & \cdots  & {H}_{1B}\\ \cdots  &  &  &  & \\ {H}_{BA} & {H}_{B1} & {H}_{B1} & \cdots  & {H}_{BB}\end{array})$$

The diagonal elements are

12 $${H}_{jj}={}_{{\bf{k}}}\langle j|\hat{H}{|j\rangle }_{{\bf{k}}}={E}_{C}-{e}^{i\frac{\pi }{3}j}{t}_{C}-{e}^{-i\frac{\pi }{3}j}{t}_{C}$$

13 $${H}_{AA}={}_{{\bf{k}}}\langle A|\hat{H}{|A\rangle }_{{\bf{k}}}={H}_{BB}={}_{{\bf{k}}}\langle B|\hat{H}{|B\rangle }_{{\bf{k}}}={E}_{Si}$$

Refer to the off-diagonal elements, due to the couplings among six C ring atoms having been considered, ${H}_{jj^{\prime} }$($j\ne j^{\prime}$) are all zero; and because the Si atoms at A and B are not neighbors, ${H}_{AB}$ is zero. So the non-zero elements of off-diagonal elements are only ${H}_{jA}$ and ${H}_{jB}$ as well as their conjugates. The non-zero elements are listed in Table 2, where ${\bf{a}}=a{\bf{i}},\,{\bf{b}}=a(-\frac{1}{2}{\bf{i}}+\frac{\sqrt{3}}{2}{\bf{j}})$ (Fig. 1(a)). These deduction procedures are similar to the case of h-SiC^25^. From Table 2, for ${H}_{jj}$, there are four different values corresponding to the four eigenvalues of $|{\phi }_{C-j}\rangle$, independent of Bloch wave vector **k**. Among the four values of ${H}_{jj}$, ${H}_{22}$ and ${H}_{44}$ are degenerated at ${E}_{C}+{t}_{C}$, and ${H}_{11}$ and ${H}_{55}$ are degenerated at ${E}_{C}-{t}_{C}$.

**Table 2:** *H* matrix elements.

*j*	${H}_{jj}$	${H}_{jA}$	${H}_{jA}$ at the K point	${H}_{jA}$ at the Γ point	${H}_{jB}$	${H}_{jB}$ at the K point,	${H}_{jB}$ at the Γ point
3	${E}_{C}+2{t}_{C}$	$\frac{-t}{\sqrt{6}}(1+{e}^{i{\bf{k}}\cdot {\bf{a}}}+{e}^{-i{\bf{k}}\cdot {\bf{b}}})$	0	$-\sqrt{\frac{3}{2}}t$	$\frac{t}{\sqrt{6}}(1+{e}^{-i{\bf{k}}\cdot {\bf{a}}}+{e}^{i{\bf{k}}\cdot {\bf{b}}})$	0	$\sqrt{\frac{3}{2}}t$
4	${E}_{C}+{t}_{C}$	$\frac{-t}{\sqrt{6}}(1+{e}^{i(-\frac{2\pi }{3}+{\bf{k}}\cdot {\bf{a}})}+{e}^{i(\frac{2\pi }{3}-{\bf{k}}\cdot {\bf{b}})})$	0	0	$\frac{-t}{\sqrt{6}}(1+{e}^{-i(\frac{2\pi }{3}+{\bf{k}}\cdot {\bf{a}})}+{e}^{i(\frac{2\pi }{3}+{\bf{k}}\cdot {\bf{b}})})$	$-\sqrt{\frac{3}{2}}t$	0
2	${E}_{C}+{t}_{C}$	$\frac{-t}{\sqrt{6}}(1+{e}^{i(\frac{2\pi }{3}+{\bf{k}}\cdot {\bf{a}})}+{e}^{i(-\frac{2\pi }{3}-{\bf{k}}\cdot {\bf{b}})})$	$-\sqrt{\frac{3}{2}}t$	0	$\frac{-t}{\sqrt{6}}(1+{e}^{-i(-\frac{2\pi }{3}+{\bf{k}}\cdot {\bf{a}})}+{e}^{i(-\frac{2\pi }{3}+{\bf{k}}\cdot {\bf{b}})})$	0	0
5	${E}_{C}-{t}_{C}$	$\frac{-t}{\sqrt{6}}(1+{e}^{i(\frac{2\pi }{3}+{\bf{k}}\cdot {\bf{a}})}+{e}^{i(-\frac{2\pi }{3}-{\bf{k}}\cdot {\bf{b}})})$	$-\sqrt{\frac{3}{2}}t$	0	$\frac{-t}{\sqrt{6}}(-1+{e}^{-i(\frac{\pi }{3}+{\bf{k}}\cdot {\bf{a}})}+{e}^{i(\frac{\pi }{3}+{\bf{k}}\cdot {\bf{b}})})$	0	0
1	${E}_{C}-{t}_{C}$	$\frac{-t}{\sqrt{6}}(1+{e}^{i(-\frac{2\pi }{3}+{\bf{k}}\cdot {\bf{a}})}+{e}^{i(\frac{2\pi }{3}-{\bf{k}}\cdot {\bf{b}})})$	0	0	$\frac{-t}{\sqrt{6}}(-1+{e}^{-i(-\frac{\pi }{3}+{\bf{k}}\cdot {\bf{a}})}+{e}^{i(-\frac{\pi }{3}+{\bf{k}}\cdot {\bf{b}})})$	$\sqrt{\frac{3}{2}}t$	0
6	${E}_{C}-2{t}_{C}$	$\frac{-t}{\sqrt{6}}(1+{e}^{i{\bf{k}}\cdot {\bf{a}}}+{e}^{-i{\bf{k}}\cdot {\bf{b}}})$	0	$-\sqrt{\frac{3}{2}}t$	$\frac{-t}{\sqrt{6}}(1+{e}^{-i{\bf{k}}\cdot {\bf{a}}}+{e}^{i{\bf{k}}\cdot {\bf{b}}})$	0	$-\sqrt{\frac{3}{2}}t$

Now we discuss the values of the elements of matrix $H({\bf{k}})$ at the K $(\frac{-2\pi }{3a}{\bf{i}}+\frac{2\pi }{\sqrt{3}a}{\bf{j}})$ point in Brillouin zone [Fig. 1(c)] listed in Table 2. At the K point, some of the elements ${H}_{jA}$ and ${H}_{jB}$ are zeros. So we can divide the basis vectors in Eq. (10) into three groups so that the couplings at the K point only exist between the vectors from the same groups but not between the vectors from different groups:

1. $${|A\rangle }_{{\bf{k}}},\,{|2\rangle }_{{\bf{k}}},\,{|5\rangle }_{{\bf{k}}};$$
2. $${|B\rangle }_{{\bf{k}}},\,{|4\rangle }_{{\bf{k}}},\,{|1\rangle }_{{\bf{k}}}$$
3. $${|3\rangle }_{{\bf{k}}},\,{|6\rangle }_{{\bf{k}}}$$

If the vector ${|1\rangle }_{{\bf{k}}}$ from the second group changes to $-{|1\rangle }_{{\bf{k}}}$, the matrix of the second group is the conjugate of the matrix of the first group in total Brillouin zone. So after diagonalization of the first and second group respectively, three pairs of energy bands can be acquired, each pair of which are equal in total Brillouin zone, and the middle pair are located near the Fermi surface. Referring to the third group, because there is little coupling between ${|3\rangle }_{{\bf{k}}}$ and ${|6\rangle }_{{\bf{k}}}$ for any wave vector **k**, the two energy levels ${E}_{C}+2{t}_{C}$ and ${E}_{C}-2{t}_{C}$ remain unchanged in the total Brillouin zone when only considering the couplings within each group.

**Figure 4:**
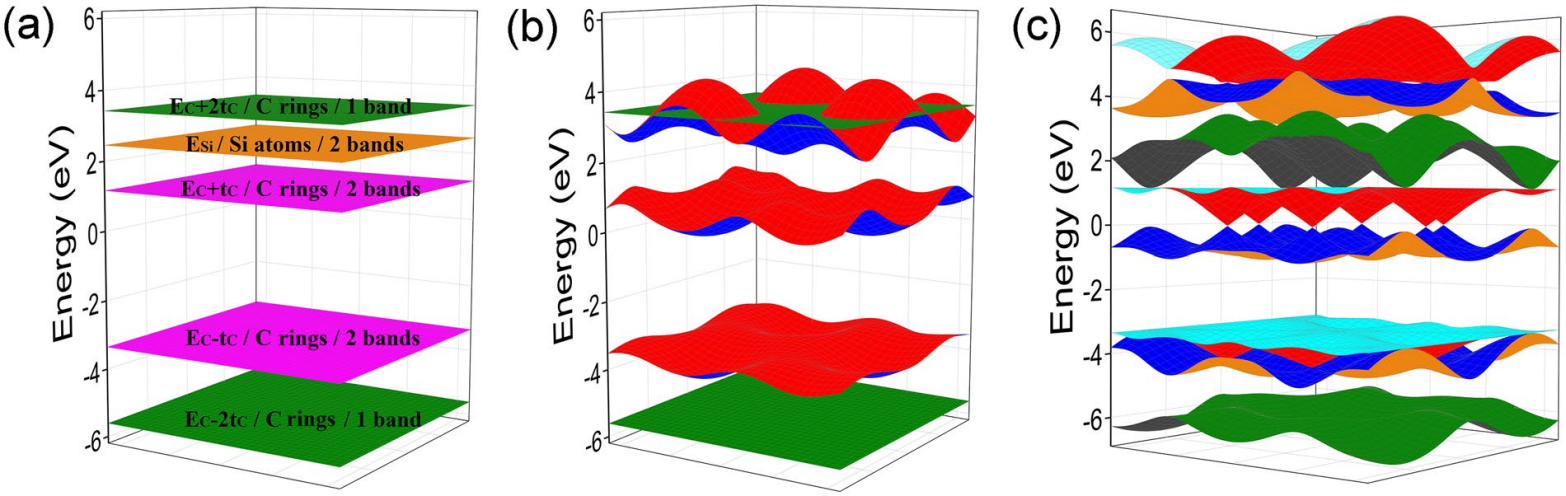
3D band structure of g-SiC_3_ from TB. (**a**) The couplings between the wave functions from same or different groups are all not considered. (**b**) Only the couplings between the wave functions from same groups are considered. (**c**) The couplings between the wave functions from same and different groups are all considered.
- The eight energy states can be divided into three groups as mentioned above. And we only consider the intra-group couplings ignoring inter-group couplings in total Brillouin zone. Then after diagonalization, both of the first and the second group generate three bands making up three pairs of bands, among which, each pair are equal in total Brillouin zone, and the middle pair lie around the Fermi surface. As for the third group, two flat bands will be acquired due to little couplings between these two states. This process is shown in Fig. 4(b).
- The inter-group couplings not considered above are included at this step. This makes the band gap to be generated except for the K points where no inter-group couplings exist. So the bands maintain touching at the K points and are separated in the other zones, resulting in the formation of DCs bands. This process is shown in Fig. 4(c).

On the basis of analysis above, we divide the formation of DCs band structure into the following three steps conceptually to understand the origin of DCs band structure:

- First, the couplings among the six C ring atoms generate six energy states, where two pairs of energy states are degenerated at ${E}_{C}+{t}_{C}$ and ${E}_{C}-{t}_{C}$, respectively, and the other two energy states are located at ${E}_{C}+2{t}_{C}$ and ${E}_{C}-2{t}_{C}$, respectively. Including the two energy states of Si atoms degenerated at ${E}_{Si}$, there are eight energy states in total. This process is shown in Fig. 4(a).

On the basis of “ring coupling” mechanism, changing the TB parameters *E*_*C*_, *E*_*Si*_, *t*_*C-C*_, and *t*_*C-Si*_ does not influence the formation of DC band structure. So this DC band structure is robust to change vertex element or ring element into other elements. This conclusion can be verified by the calculation of g-Si_3_C, g-GeC_3_, g-Ge_3_C, g-GeSi_3_, and g-Ge_3_Si later in this work. If the onsite energies of the two vertex atoms are not equal due to different types of vertex atoms, the three pairs of bands generated by the couplings within the first group and the second group would possess different values within each pair of bands at the K point, leading to a semiconducting system. This explains why SiC_7_ is a semiconductor^29^.

### Conditions of g-SiC_3_-like systems possessing self-doped band structure

**Figure 5:**
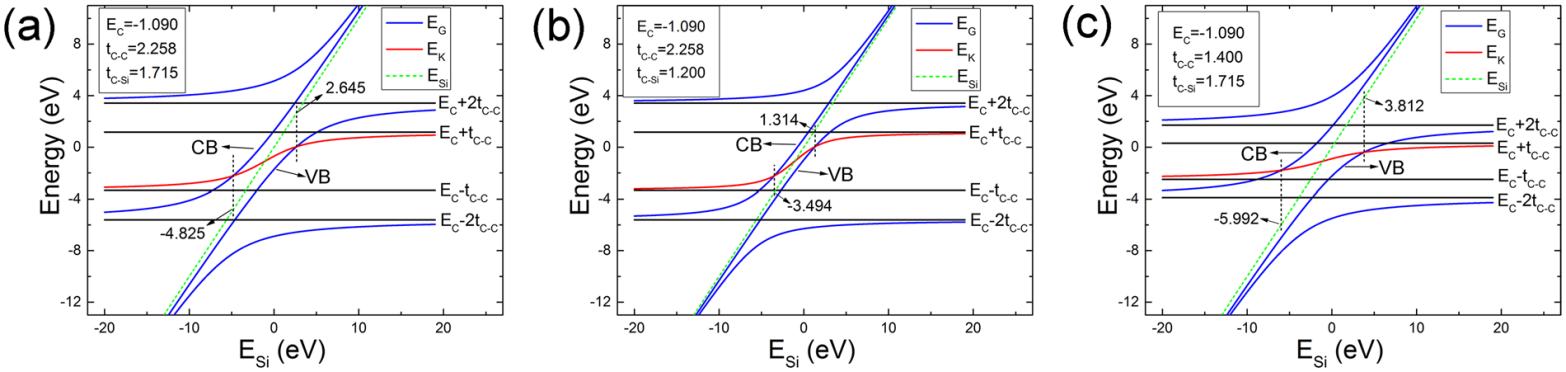
The values of band at the Γ and the K point of g-SiC_3_ with scanning *E*_*Si*_ and other parameters unchanged. The black lines express the four levels from the couplings of the hexagon C ring. The green lines with the equal horizontal coordinate and vertical coordinate express the parameter *E*_*Si*_. The blue lines express the four values of bands at the Γ point which are the eigenvalues of the Hamilton submatrix with the vectors in Eq. (14) as basis set at the Γ point. The red lines express the value of Dirac point which is the middle eigenvalue of the Hamilton submatrix with the vectors of the first group as basis set at the K point. (**a**) The TB parameters unchanged compared to g-SiC_3_. (**b**) Decreasing the hopping energy between C and Si (*t*_*C-Si*_) with hopping energy between C and C (*t*_*C-C*_) as well as onsite energy of C (*E*_*C*_) unchanged compared to g-SiC_3_. (**c**) Decreasing the hopping energy between C and C (*t*_*C-C*_) with hopping energy between C and Si (*t*_*C-Si*_) as well as the onsite energy of C (*E*_*C*_) unchanged compared to g-SiC_3_.

From the band structure of g-SiC_3_ [Fig. 2(a)], near Fermi surface, the energy value of valence band (VB) at the Γ point is very close to the energy value of the K point where DC appears (DC point). If the energy value of VB at the Γ point is slightly higher than DC point, the DC point would be slightly lower than Fermi surface, forming the so-called self-doped system^4, 30^. So it is important to compare the value of VB or CB (if the value of CB at the Γ point is lower than the DC point, the DC point will be higher than Fermi surface) at the Γ point with DC point. From discussion above, the energy value of DC point is the middle eigenvalue of the matrix of the first group (or the second group) at the K point. While, from Table 2, at the Γ point, the couplings only exist among the four wave functions

14 $${|3\rangle }_{{\bf{k}}},{|6\rangle }_{{\bf{k}}},{|A\rangle }_{{\bf{k}}}\,{\rm{and}}\,{|B\rangle }_{{\bf{k}}}$$

corresponding to the energy levels ${E}_{C}+2{t}_{C}$, ${E}_{C}-2{t}_{C}$, ${E}_{Si}$, and ${E}_{Si}$, respectively. To acquire the values of VB and CB at the Γ point, the Hamiltonian matrix with the vectors in Eq. (14) as basis set is diagonalized at the Γ point with scanning ${E}_{Si}$ and other parameters remaining unchanged. The result is shownin Fig. 5(a). And for comparing the values of VB and CB at the Γ point with the DC point, we calculated the values of DC point with scanning ${E}_{Si}$ without changing the other parameters as shown in Fig. 5(a). We discuss the results as the follows.

1. When ${E}_{Si}$ is near ${E}_{C}$ (−4.825 eV < ${E}_{Si}$ < 2.645 eV), the value of DC point is higher than the value of VB at the Γ and lower than the value of CB at Γ, so the DC point exists on the Fermi surface, and this system is a DC system.
2. When ${E}_{Si}$ is far from ${E}_{C}$ (${E}_{Si}$ < −4.825 eV or ${E}_{Si}$ > 2.645 eV), the DC point deviated from the Fermi surface. Specially, when ${E}_{Si}$ < −4.825 eV, the value of CB at the Γ is lower than the value of DC point, leading to DC point higher than Fermi surface; while when ${E}_{Si}$ > 2.645 eV, the value of VB at the Γ is higher than the value of DC point, leading to DC point lower than Fermi surface.
3. When ${E}_{Si}$ < −4.825 eV or ${E}_{Si}$ > 2.645 eV, but ${E}_{Si}$ is very close to −4.825 eV or 2.645 eV, the DC point deviated only slightly from the Fermi surface, leading to the formation of a self-doped system.

So, increasing the difference between the onsite energies of vertex atoms and ring atoms change the systems into self-doped systems. The calculations for g-GeC_3_ and g-Ge_3_C later in this paper support this conclusion.

To examine the influence of hoping energy on the formation of self-doped systems, we changed the hoping energy ${t}_{C-Si}$ (${t}_{C-C}$), and performed the same calculations required in Fig. 5(a) to acquire Fig. 5(b) [Fig. 5(c)].

From Fig. 5(b), reducing ${t}_{C-Si}$, from 1.715 to 1.2 eV, with ${E}_{C}$ and ${t}_{C-C}$ unchanged, shrinks the range of ${E}_{Si}$, from [−4.825, 2.645] eV to [−3.494, 1.314] eV, in which the DC point is located on the Fermi level. While, from Fig. 5(c), reducing ${t}_{C-C}$, from 2.258 to 1.4 eV, with ${E}_{C}$ and ${t}_{C-Si}$ unchanged, enlarges the range of ${E}_{Si}$, from [−4.825, 2.645] eV to [−5.992, 3.812] eV, in which the DC point is located on the Fermi level. So, decreasing ${t}_{C-Si}$ and increasing ${t}_{C-C}$ may change the system into self-doped system.

**Figure 6:**
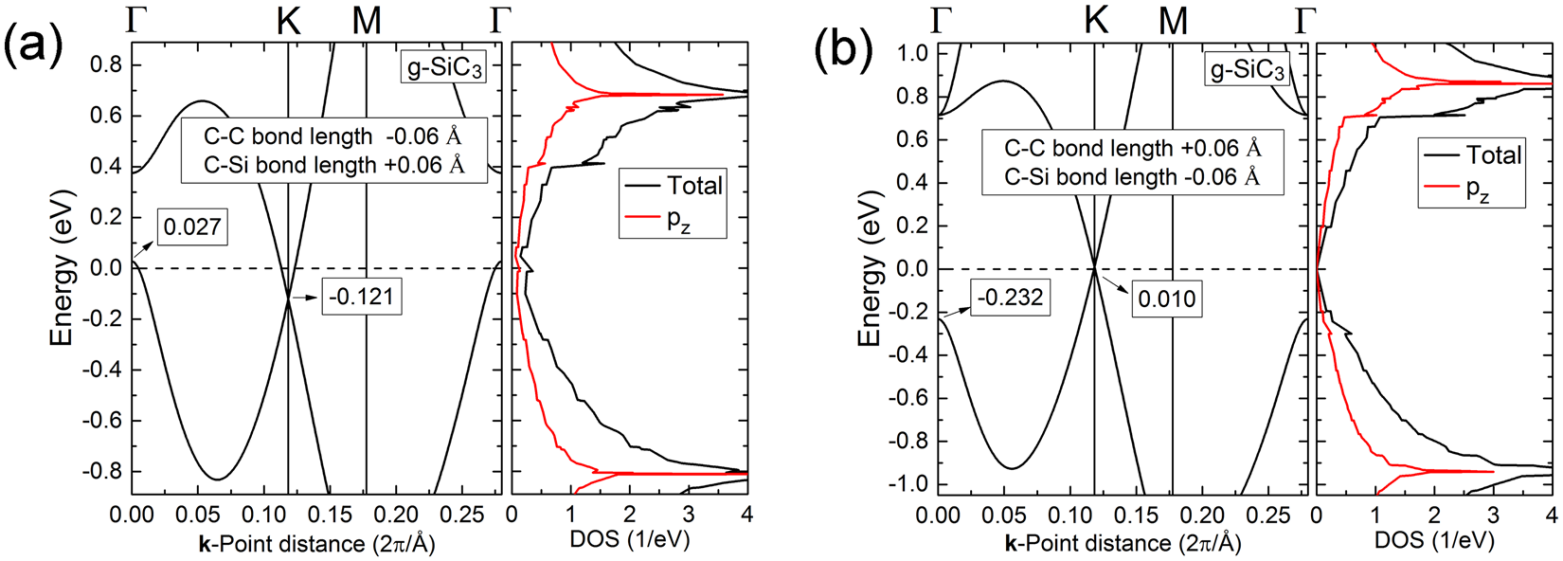
Band structures and DOS of g-SiC_3_ with C-C and C-Si bond length changed and lattice parameter unchanged. (**a**) C-C bond length is decreased with 0.06 Å and C-Si bond length is increased with 0.06 Å with lattice parameter unchanged. (**b**) C-C bond length is increased with 0.06 Å and C-Si bond length is decreased with 0.06 Å with lattice parameter unchanged.

Increasing (decreasing) bond length can mimic the decreasing (increasing) of hopping energy, so increasing the C-Si bond length and/or decreasing the C-C bond length may change g-SiC_3_ into self-doped system, while increasing the C-C bond length and/or decreasing the C-Si bond length increase the difference between the value of Dirac point and the value of VB at the Γ point compared with the equilibrium system. We decreased (increased) the C-C bond length with 0.06 Å and increased (decreased) the C-Si bond length with 0.06 Å, keeping the lattice parameter unchanged; then calculated their band structures by DFT (Fig. 6). From Fig. 6, we found that: (1) When the C-C bond length is decreased by 0.06 Å and C-Si bond length is increased by 0.06 Å, with lattice parameter unchanged, the value of Dirac point (−0.121 eV) is lower than the value of VB at the Γ point (0.027 eV), forming a self-doped system [Fig. 6(a)]. (2) When the C-C bond length is increased with 0.06 Å and C-Si bond length is decreased by 0.06 Å, with lattice parameter unchanged, the difference (0.242 eV) between the value of Dirac point (0.010 eV) and the value of VB at the Γ point (−0.232 eV) increases [Fig. 6(b)] compared with the equilibrium system. (For the equilibrium system, the value of Dirac point is −0.027 eV, the value of valence at the Γ point is −0.067 eV, and the difference is 0.040 eV. [Fig. 2(a)]) So the DF calculations support our TB analysis above, and we may tune the bond length of g-SiC_3_ to change g-SiC_3_ into a self-doped system by depositing the monolayers on appropriate substrates. Thermal vibrations around equilibrium atom positions are not expected to affect the self-doping behavior due to random features of bond length changes.

### Band structure of g-Si_3_C

**Figure 7:**
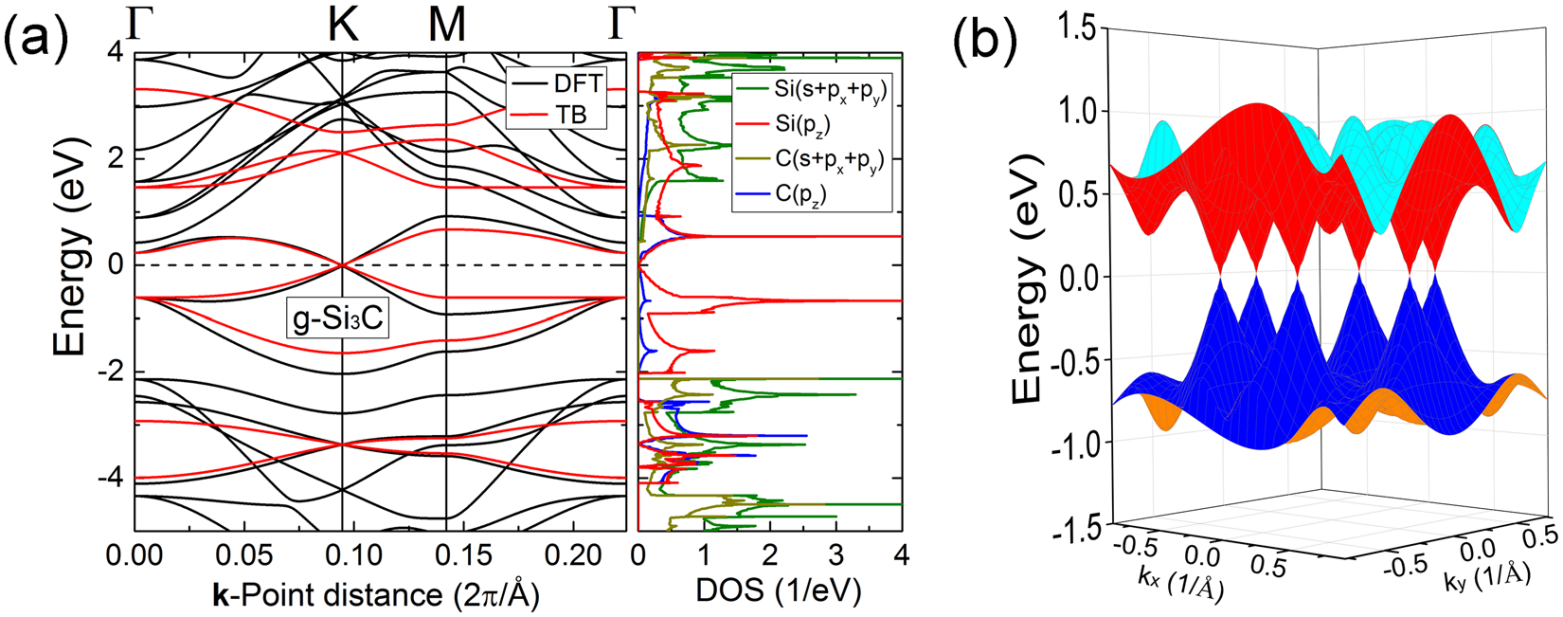
(**a**) Band structure (left) and DOS (right) of g-Si_3_C. For the band structure, the black lines are the results calculated by DFT and the red lines are the results calculated by TB. (**b**) 3D band structure of g-Si_3_C calculated by DFT.

g-Si_3_C (Fig. 1(b)), possessing similar atomic structure as g-SiC_3_ [Fig. 1(a)], also displays DCs in band structure (Fig. 7) due to “ring coupling” mechanism referring to the couplings of six Si ring atoms. The group velocity of g-Si_3_C near Fermi surface is listed in Table 1 after averaged over electrons and holes as well as different directions. The electron/hole group velocity of g-Si_3_C is lower than that of graphene or g-SiC_3_ and is similar to that of silicene. These results are related to the transport properties discussed later.

**Figure 8:**
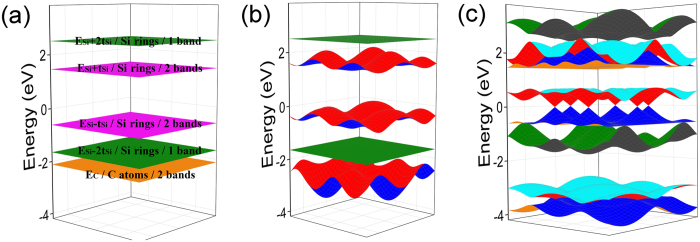
3D band structure of g-Si_3_C by TB. (**a**) The couplings between the wave functions from same or different groups are all not considered. (**b**) Only the couplings between the wave functions from same groups are considered. (**c**) The couplings between the wave functions from same and different groups are all considered.

Figure 8 shows the formation process of DCs band structure of g-Si_3_C similar to the formation process of DCs band structure of g-SiC_3_ (Fig. 4). The TB parameters are obtained by fitting DFT results: the onsite energies of C and Si are *E*_*C*_ = −2.113 eV and *E*_*Si*_ = 0.428 eV, respectively; The hopping energies of Si-Si and C-Si are *t*_*Si-Si*_ = 1.037 eV and *t*_*C-Si*_ = 1.212 eV, respectively.

Figure 8(a) shows differences opposite to Fig. 4(a): for Fig. 8(a) which is the “band structure” of g-Si_3_C, the band from vertex A (or B) lie out of the “other four bands” (the four energy levels from the coupling of the six same type atoms in a ring), while for Fig. 4(a) which is the “band structure” of g-SiC_3_, the band from vertex A (or B) lie among the “other four bands”. This can be explained as follows: because the C-C coupling is stronger than the Si-Si coupling (*t*_*C-C*_ > *t*_*Si-Si*_), the differences between the highest band and the lowest band of the “other four band” for g-Si_3_C is smaller than g-SiC_3_, which results to the band from vertex A (or B) for g-Si_3_C laying out of the “other four bands”.

### Atomic structures and band structures of g-GeC_3_, g-Ge_3_C, g-GeSi_3_, and g-Ge_3_Si

**Figure 9:**
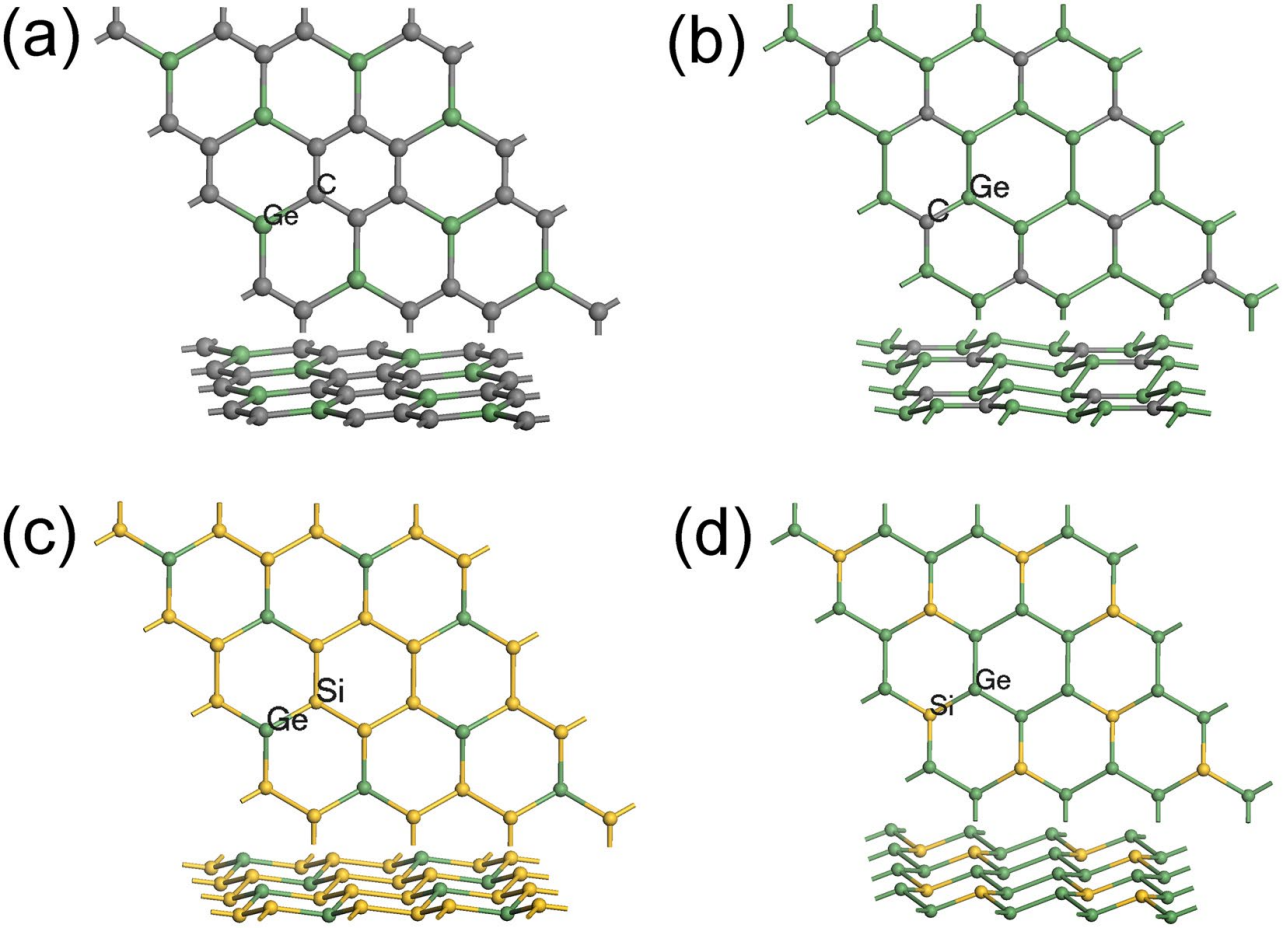
Atomic structures of (**a**) g-GeC_3_, (**b**) g-Ge_3_C, (**c**) g-GeSi_3_, and (**d**) g-Ge_3_Si.

Similar to g-SiC_3_ and g-Si_3_C, substituting Si or C with Ge from the same main group in the periodic table, we constructed the binary models of g-GeC_3_, g-Ge_3_C, g-GeSi_3_, and g-Ge_3_Si. Their atomic structures optimized by DFT are shown in Fig. 9. Their atomic structure parameters and formation energy are listed in Table 3. For the purpose of comparison, we optimized the geometry structure of germanene. Due to silicene and germanene preferring to sp^3^ hybridization, g-Ge_3_C, g-GeSi_3_, and g-Ge_3_Si are all buckled with non-planar structures. While, g-GeC_3_ is a planar structure with all atoms in a plane because carbon prefer to sp^2^ hybridization and this structure consists of more carbon atoms than g-Ge_3_C. These results are similar to the study of Zhao *et al*. except for the g-Ge_3_C (a planar structure in their studies)^30^. As shown in Tables 1 and 3, the formation energy ${E}_{f}$ of g-GeC_3_, g-Ge_3_C, g-GeSi_3_, and g-Ge_3_Si decreases gradually, which can be understood by the fact that the formation energy ${E}_{f}$ decreases in the order of graphene (9.23 eV), silicene (4.77 eV) and germanene (4.03 eV) and the energy ${E}_{f}$ of silicene is very close to germanene.

**Table 3:** Bond lengths *l* (Å), lattice parameters *a* (Å), size of buckle *d*_z_ (Å) and formation energies per atom [${E}_{f}$ and ${E}_{f^{\prime} }$ (eV)] of g-GeC_3_, g-Ge_3_C, g-GeSi_3_, g-Ge_3_Si, and germanene.

A_x_B_y_	*l*_A-A_/*l*_B-B_	*l*_A-B_	*a*	*d*_z_	${{\bf{E}}}_{{\bf{f}}}$	${{\bf{E}}}_{{\bf{f}}^{\prime} }$
g-GeC_3_	1.43	1.88	5.74	0	7.41	−0.52
g-Ge_3_C	2.42	1.91	7.30	0.67	4.87	−0.46
g-GeSi_3_	2.28	2.34	7.80	0.57	4.58	−0.01
g-Ge_3_Si	2.42	2.36	7.98	0.65	4.20	−0.02
Germanene	2.43		4.04	0.68	4.03	

**Figure 10:**
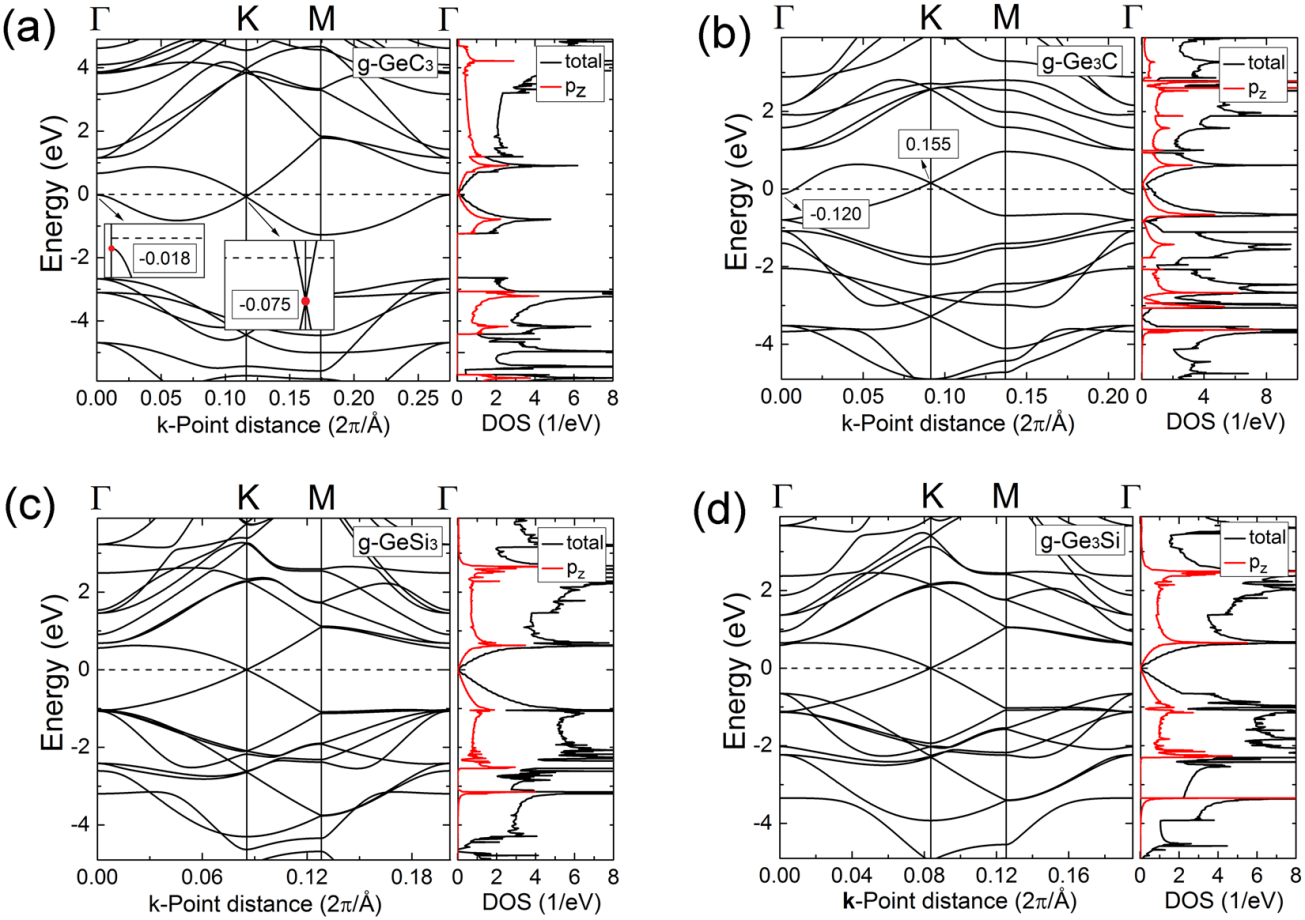
Band structures and DOS of (**a**) g-GeC_3_, (**b**) g-Ge_3_C, (**c**) g-GeSi_3_, and (**d**) g-Ge_3_Si.

The g-GeC_3_, g-Ge_3_C, g-GeSi_3_, and g-Ge_3_Si systems also possess DCs band structures (Fig. 10) due to possessing similar atomic structures as g-SiC_3_ and g-Si_3_C, as well as all elements belong to IV group. A small difference is that the Dirac points of g-GeC_3_ and g-Ge_3_C deviate slightly from Fermi surface, leading to the formation of self-doped systems. Specifically, as for g-GeC_3_, because the value of VB at the Γ point is higher than the value of Dirac point, the DC point is lower than Fermi surface. According to the discussions above, it is understood that the difference between the onsite energies of Ge and C atom is larger than that between Si and C atom as well as the hopping energy between Ge and C atom is smaller than that between Si and C atom. And referring to g-Ge_3_C, the value of CB at the Γ point is lower than the Dirac point, so the Dirac point is higher than Fermi surface. Because the atomic structure of g-Ge_3_C is buckled, the p_z_ orbitals may be coupled to the other orbitals, and the band near Fermi surface may include the other orbitals except for the p_z_ orbitals, leading to the formation of self-doped system. From the band structure of planar g-Ge_3_C calculated by Zhao *et al*.^30^, there is a band from non-p_z_ orbitals near Fermi surface, consistent with our analysis. These results agree with the studies of Zhao *et al*.^30^.

When the C atom of g-Si_3_C is substituted by B, N, Al, or P, the atomic model structures of XSi_3_ (X = B, N, Al, or P) can be constructed. When the Si atom of g-SiC_3_ is substituted by B, BC_3_ can be constructed. According to the analyses above, these structures should also possess DC band structures. However, because the numbers of the valence electrons of these structures are different from g-SiC_3_ or g-Si_3_C, the DCs of these structures are either above or under the Fermi surface. Previous studies support this discussion^48, 49^.

### Electron transport properties of g-SiC_3_ and g-Si_3_C nanoribbons

For the potential nanoelectronic device applications, we calculated directly the electron transport properties of g-SiC_3_ and g-Si_3_C nanoribbons.

**Figure 11:**
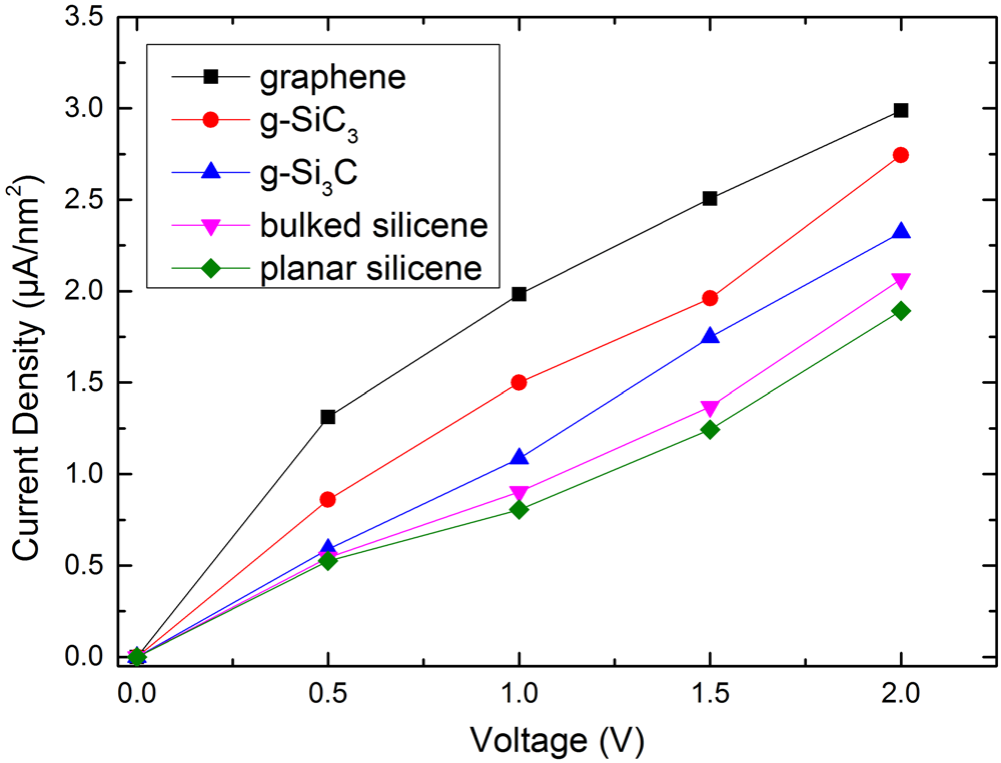
Current density versus voltage relations of graphene (black square), g-SiC_3_ (red circle), g-Si_3_C (blue triangle), bulked silicene (pink down triangle), planar silicene (green diamond) under bias voltages of 0.5 V, 1.0 V, 1.5 V, and 2.0 V.

The current density versus voltage curves were calculated and shown in Fig. 11 for the lead-molecule-lead junctions. Here we showed the current density, dividing the current by the surface area of electrode. The current density and voltage have nearly linear relationship over the bias voltages ranging from 0 to 2.0 V. We found that the current of g-SiC_3_ is larger than g-Si_3_C, both of which are smaller than graphene but larger than silicenes in both bulked and planar forms. It is known that graphene has larger electron conductance than silicene^50^. The studied binary monolayers have conductance between graphene and silicene and the conductance increases as the C concentration increases (Figure S5 in Supplementary Information). Table 4. lists the electron conductance of the systems under various bias voltages. These electron transport results are consistent with the electron/hole group velocities calculated from the band structures shown earlier.

**Table 4:** Electron conductance of graphene, g-SiC_3_, g-Si_3_C, bulked silicene, and planar silicene under various bias voltages.

Conductance(*μS*)	0.5 V	1 V	1.5 V	2 V
Graphene	9.48	7.61	6.49	5.73
g-SiC_3_	5.96	5.74	5.13	5.32
g-Si_3_C	4.54	4.21	4.49	4.48
Bulked silicene	4.18	3.62	3.52	3.99
Planar silicene	4.02	3.18	3.25	3.64

## Conclusions

In this work we proposed a “ring coupling” mechanism to illustrate the formation of DCs of g-SiC_3_ and g-Si_3_C as the examples of binary monolayers AB_3_ and A_3_B (A, B = C, Si, and Ge): (1) the couplings of six C ring atoms form six new wave functions corresponding to four energy levels. The middle two energy levels are doubly degenerated, respectively. (2) The two wave functions of Si and the four wave functions corresponding to the middle two doubly degenerated levels are divided into two groups; each group contains one wave function of Si and two wave functions each from the two different doubly degenerated wave functions. The intra-group coupling of each group forms three bands, and there are six bands in total from these two groups. The six bands make up three pairs, and each pair are equal at the K point. The rest two of the six functions from the couplings of six C ring atoms form two flat bands (they are the third group). (3) After considering the inter-group couplings among the three groups, the gap is formed. However, there are no inter-group couplings at the K point where the bands remain contact, leading to the formation of DCs.

Based on this “ring coupling” mechanism, the possible methods changing the g-SiC_3_-like monolayers into self-doped systems are discussed: (a) Increasing the difference between the onsite energies of ring atom and vertex atom, (b) decreasing the hopping energies between the ring atom and vertex atom. (c) increasing the hopping energy between the two ring atoms.

The “ring coupling” mechanism proposed in this work is applicable to 2D DC materials possessing ring patterns. We previously also studied other typical 2D structures. Specifically, we used the “pair coupling” mechanism to explain DC formation in 2D materials with paired atoms, e.g. t1-SiC^25^. Moreover, we proposed the “triple coupling” mechanism to understand DC formation in α-grahynes where triple atom-chains were coupled first^39^. The “ring coupling”, “pair coupling”, and “triple coupling” mechanisms share the similar methodology but account for various arrangement patterns in understanding the general mechanism of Dirac cone formation in 2D materials, thus they can be unified into a more general framework called “divide-and-couple”, which can be applied to illustrate the origins of Dirac cone formation in other Fermi Dirac systems.

### Method and computational details

In this work, the DFT calculations were carried out using the Vienna *ab initio* simulation package (VASP)^51, 52^. The exchange-correlation function and pseudopotentials adopted the form of Perdew-Burke-Ernzerh (PBE) within a generalized gradient approximation (GGA)^53^ and the projector augmented-wave (PAW) method^54^ respectively. For binary 2D systems, we adopted 700 eV energy cutoff for the expansion of plane wave basis set and (7 × 7 × 1) for Monkhorst-Pack sampling, leading to convergence of 0.001 eV. For unitary 2D systems, the (17 × 17 × 1) Monkhorst-Pack grid was used. The SCF calculations converge to 5.0 × 10^−7^ eV/atom, while the geometry optimizations converge to 5.0 × 10^−6^ eV/atom using conjugated gradient method. The QMD calculations were carried out with a 700 eV energy cutoff, a (5 × 5 × 1) Monkhorst-Pack k-point sampling, and the SCF tolerance 1.25 × 10^−7^ eV/atom. The vacuum region among layers is longer than 15 Å to avoid the influences among periodic images.

To evaluate the electron transport properties for their potential applications as electronic devices, we calculated the current-voltage (I-V) characteristics, electron transmission spectrum, and density of states of g-SiC_3_ and g-Si_3_C, compared with graphene and silicene in both bulked and planar forms using *ab initio* modeling package nanodcal^55, 56^. Figure S4(a) in the Supplementary Information illustrates the lead-molecule-lead junction with the semi-infinite Au lead. We first optimized the molecule-electrode distances using the DMol^3^ program. The Perdew-Burke-Ernzerhof (PBE) functional under General Gradient Approximation (GGA) was adopted with double-ζ polarization basis set and DFT Semi-core pseudopotentials^57, 58^. T = 300 K was used for the Fermi-Dirac distribution around Fermi level throughout the work of this section. Figure S4(b–f) shows the configurations of the optimized junctions.

## Electronic supplementary material

Supplementary Information

## References

[1] Novoselov KS (2004). Electric field effect in atomically thin carbon films. Science.

[2] Baughman, R. H., Eckhardt, H. & Kertesz, M. Structure‐property predictions for new planar forms of carbon: Layered phases containing sp^2^ and sp atoms. *J. Chem. Phys*. **87**, 6687-6699 (1987).

[3] Li GX (2010). Architecture of graphdiyne nanoscale films. Chem. Commun..

[4] Malko D, Neiss C, Viñes F, Görling A (2012). Competition for graphene: Graphynes with direction-dependent Dirac cones. Phys. Rev. Lett..

[5] Qian XM (2015). Self-catalyzed growth of large-area nanofilms of two-dimensional carbon. Sci. Rep..

[6] Zhao JJ (2016). Rise of silicene: A competitive 2D material. Prog. Mater. Sci..

[7] Aufray B (2010). Graphene-like silicon nanoribbons on Ag(110): A possible formation of silicene. Appl. Phys. Lett..

[8] Cahangirov S, Topsakal M, Aktürk E, Şahin H, Ciraci S (2009). Two- and one-dimensional honeycomb structures of silicon and germanium. Phys. Rev. Lett..

[9] Liu H (2014). Phosphorene: An unexplored 2D semiconductor with a high hole mobility. ACS Nano.

[10] Guan J, Zhu Z, Tománek D (2014). Phase coexistence and metal-insulator transition in few-layer phosphorene: A computational study. Phys. Rev. Lett..

[11] Tang H, Ismail-Beigi S (2007). Novel precursors for boron nanotubes: The competition of two-center and three-center bonding in boron sheets. Phys. Rev. Lett..

[12] Peng B (2016). The electronic, optical, and thermodynamic properties of borophene from first-principles calculations. J. Mater. Chem. C.

[13] Mannix AJ (2015). Synthesis of borophenes: Anisotropic, two-dimensional boron polymorphs. Science.

[14] Coleman JN (2011). Two-dimensional nanosheets produced by liquid exfoliation of layered materials. Science.

[15] Sun X-H (2002). Formation of silicon carbide nanotubes and nanowires via reaction of silicon (from disproportionation of silicon monoxide) with carbon nanotubes. J. Am. Chem. Soc..

[16] Xie ZF, Tao DL, Wang JQ (2007). Synthesis of silicon carbide nanotubes by chemical vapor deposition. J. Nanosci. Nanotechnol..

[17] Banerjee S, Majumder C (2013). Conformers of hydrogenated SiC honeycomb structure: A first principles study. AIP Adv..

[18] Lin X (2013). Ab initio study of electronic and optical behavior of two-dimensional silicon carbide. J. Mater. Chem. C.

[19] Garcia JC, de Lima DB, Assali LVC, Justo JF (2011). Group IV graphene- and graphane-like nanosheets. J. Phys. Chem. C.

[20] Zhao K, Zhao MW, Wang ZH, Fan YC (2010). Tight-binding model for the electronic structures of SiC and BN nanoribbons. Physica E.

[21] Sun L (2008). Electronic structures of SiC nanoribbons. J. Chem. Phys..

[22] Bekaroglu E, Topsakal M, Cahangirov S, Ciraci S (2010). First-principles study of defects and adatoms in silicon carbide honeycomb structures. Phys. Rev. B.

[23] Chen XP (2016). Tunable electronic structure and enhanced optical properties in quasi-metallic hydrogenated/fluorinated SiC heterobilayer. J. Mater. Chem. C.

[24] Wang N, Tian Y, Zhao JX, Jin P (2016). CO oxidation catalyzed by silicon carbide (SiC) monolayer: A theoretical study. J. Mol. Graph. Model..

[25] Qin XM (2015). Origin of Dirac cones in SiC silagraphene: A combined density functional and tight-binding study. J. Phys. Chem. Lett..

[26] Shahrokhi M, Leonard C (2017). Tuning the band gap and optical spectra of silicon-doped graphene: Many-body effects and excitonic states. J. Alloy. Compd..

[27] Li YF, Li FY, Zhou Z, Chen ZF (2011). SiC_2_ silagraphene and its one-dimensional derivatives: Where planar tetracoordinate silicon happens. J. Am. Chem. Soc..

[28] Zhang CZ, Zhang SH, Wang Q (2016). Bonding-restricted structure search for novel 2D materials with dispersed C_2_ dimers. Sci. Rep..

[29] Dong HL (2016). SiC_7_ silagraphene: a novel donor material with extraordinary sunlight absorption. Nanoscale.

[30] Zhao MW, Zhang RQ (2014). Two-dimensional topological insulators with binary honeycomb lattices: SiC_3_ silagraphene and its analogs. Phys. Rev. B.

[31] Ding Y, Wang YL (2014). Geometric and electronic structures of two-dimensional SiC_3_ compound. J. Phys. Chem. C.

[32] Semenoff, G. W. Condensed-matter simulation of a three-dimensional anomaly. *Phys. Rev. Lett*. **53**, 2449-2452 (1984).

[33] Castro Neto AH, Guinea F, Peres NMR, Novoselov KS, Geim AK (2009). The electronic properties of graphene. Rev. Mod. Phys..

[34] Neugebauer P, Orlita M, Faugeras C, Barra A-L, Potemski M (2009). How perfect can graphene be?. Phys. Rev. Lett..

[35] Zhao MW, Dong WZ, Wang AZ (2013). Two-dimensional carbon topological insulators superior to graphene. Sci. Rep..

[36] Zhang LZ (2015). Highly anisotropic Dirac fermions in square graphynes. J. Phys. Chem. Lett..

[37] Zhou X-F (2014). Semimetallic two-dimensional boron allotrope with massless Dirac fermions. Phys. Rev. Lett..

[38] Ding Y, Wang YL (2015). Unusual structural and electronic properties of porous silicene and germanene: insights from first-principles calculations. Nanoscale Res. Lett..

[39] Qin XM, Liu Y, Chi BQ, Zhao XL, Li XW (2016). Origins of Dirac cones and parity dependent electronic structures of α-graphyne derivatives and silagraphynes. Nanoscale.

[40] Malko D, Neiss C, Görling A (2012). Two-dimensional materials with Dirac cones: Graphynes containing heteroatoms. Phys. Rev. B.

[41] Ma YD, Dai Y, Huang BB (2013). Dirac cones in two-dimensional lattices: Janugraphene and chlorographene. J. Phys. Chem. Lett..

[42] Sun ML, Wang S, Yu J, Tang WC (2017). Hydrogenated and halogenated blue phosphorene as Dirac materials: A first principles study. Appl. Surf. Sci..

[43] Wang ZF, Liu Z, Liu F (2013). Quantum anomalous Hall effect in 2D organic topological insulators. Phys. Rev. Lett..

[44] Wei L, Zhang XM, Zhao MW (2016). Spin-polarized Dirac cones and topological nontriviality in a metal-organic framework Ni_2_C_24_S_6_H_12_. Phys. Chem. Chem. Phys..

[45] Wang JY, Deng SB, Liu ZF, Liu ZR (2015). The rare two-dimensional materials with Dirac cones. Natl. Sci. Rev..

[46] Kim BG, Choi HJ (2012). Graphyne: Hexagonal network of carbon with versatile Dirac cones. Phys. Rev. B.

[47] Huang HQ, Duan WH, Liu ZR (2013). The existence/absence of Dirac cones in graphynes. New J. Phys..

[48] Ding Y, Wang YL (2013). Density functional theory study of the silicene-like SiX and XSi_3_ (X = B, C, N, Al, P) honeycomb lattices: The various buckled structures and versatile electronic properties. J. Phys. Chem. C.

[49] Ding Y, Wang YL, Ni J (2009). Electronic structures of BC_3_ nanoribbons. Appl. Phys. Lett..

[50] Yamacli S (2014). Comparison of the electronic transport properties of metallic graphene and silicene nanoribbons. J. Nanopart. Res..

[51] Kresse G, Hafner J (1993). Ab initio molecular dynamics for open-shell transition metals. Phys. Rev. B.

[52] Kresse G, Furthmüller J (1996). Efficient iterative schemes for *ab initio* total-energy calculations using a plane-wave basis set. Phys. Rev. B.

[53] Perdew JP, Burke K, Ernzerhof M (1996). Generalized Gradient Approximation Made Simple. Phys. Rev. Lett..

[54] Kresse G, Joubert D (1999). From ultrasoft pseudopotentials to the projector augmented-wave method. Phys. Rev. B.

[55] Taylor J, Guo H, Wang J (2001). Ab initio modeling of quantum transport properties of molecular electronic devices. Phys. Rev. B.

[56] Taylor J, Guo H, Wang J (2001). Ab initio modeling of open systems: Charge transfer, electron conduction, and molecular switching of a C_60_ device. Phys. Rev. B.

[57] Delley B (1990). An all‐electron numerical method for solving the local density functional for polyatomic molecules. J. Chem. Phys..

[58] Delley B (2000). From molecules to solids with the DMol^3^ approach. J. Chem. Phys..

