# Supplementary material for: Origins of Dirac cone formation in AB3 and A3B (A, B = C, Si, and Ge) binary monolayers
Source: Sci Rep. 2017 Sep 5;7:10546. doi: 10.1038/s41598-017-10670-x (PMC5585377; doi:10.1038/s41598-017-10670-x)
Supplement: Supplementary file 1 — Supplementary Information [file 41598_2017_10670_MOESM1_ESM.pdf]

Supplementary Information

Origins of Dirac cone formation in AB_3 and A_3B (A, B= C, Si, and Ge) binary monolayers

Xuming Qin[a,b], *Yuqin Wu*[a], *Yi Liu*[a,] *, *Baoqian Chi*[a,b], *Xiaowu Li*[b,] *, *Yin Wang*[a] & *Xinluo Zhao*[a]

[a] Department of Physics, Materials Genome Institute, and International Centre for Quantum and Molecular Structures, Shanghai University, 99 Shangda Road, Shanghai 200444, P. R. China.

[b] Department of Materials Physics and Chemistry, School of Materials Science and Engineering, and Key Laboratory for Anisotropy and Texture of Materials (Ministry of Education), Northeastern University, No. 3-11 Wenhua Road, Shenyang 110819, P. R. China.

*Corresponding authors:

E-mail address: yiliu@t.shu.edu.cn (Yi Liu);

E-mail address: xwli@mail.neu.edu.cn (Xiaowu Li)

.

S1 Results of quantum molecular dynamics calculations

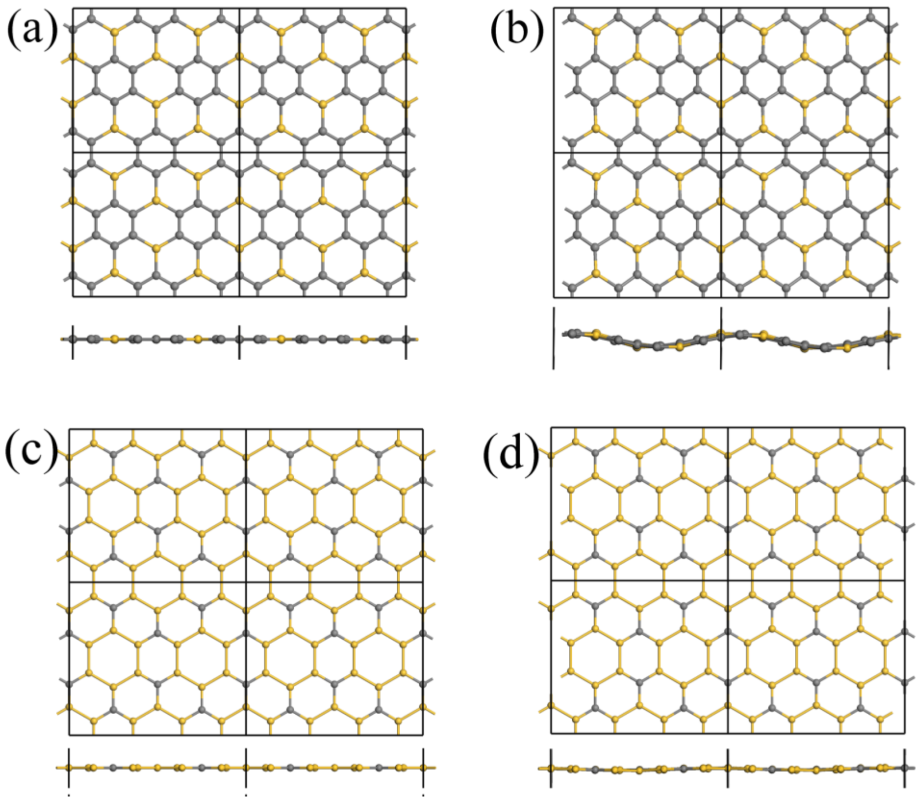

Figure S1. Atomic structures of g-SiC_3 at 0 ps (a) and 2.5 ps (b) as well as atomic structures of g-Si_3C at 0 ps (c) and 2.5 ps (d) acquired by the quantum molecular dynamics calculations at a NVT ensemble at 600 K in 32-atom cells. The upper figures are top view, and the lower figures are side view.

S2 Interfacial energy and formation energy of segregated interface models

If atomic segregation leads to separated graphene and silicene nanoribbons, the Si-C bonds form at the interface to connect the two nanoribbons dubbed the segregated interface model. The SiC_3 or Si_3C models (Figure 1 (a, b) in the manuscript) studied earlier are called the mixture models. The interface energy per bond here is represented by the energy change associated with the interface bond substitution plus the induced distortion energy compared with the undistorted pristine sheets. The formation energy of the segregated interface models depend on the interface energy and the concentration of interfaces that can be represented by the width of the nanoribbons or the separation distances between two interfaces. Here we calculated the interface energies for the segregated interface model consisting of

graphene and silicene nanoribbons (Figure S2) with and without distortion. Moreover, we show the formation energies as functions of the width of the nanoribbons in term of number of atomic layers (Figure S3).

The interface energy without distortion is the energy change by substituting one half of C-C bond in graphene and one half of Si-Si bond in silicene with a Si-C bond in SiC defined as

$$E_{\text{int}} = \frac{1}{3}E_{f-gra} + \frac{1}{3}E_{f-sil} - \frac{2}{3}E_{f-SiC} \quad \text{(S1)}$$

where E_{int} is the interface energy per Si-C bond; E_{f-gra}, E_{f-sil}, and E_{f-SiC} are the formation energies per atom relative to isolated atoms of graphene, silicene, and h-SiC with alternative Si and C arrangement. These formation energies are adopted from our previous calculations[1,2] (E_{f-SiC} is from ref. 1; E_{f-gra} and E_{f-sil} are from ref. 2). In eq. (S1) the first two terms represent the halves of C-C and Si-Si bond energies respectively, and the third term $\frac{2}{3}E_{f-SiC}$ represents the Si-C bond energy because each atom has three bonds and each bond connects two atoms.

We fully optimized the segregated interface models shown in Figure S2 and obtained the distorted interface that allows to include the distortion effects. The interface energy with distortion per Si-C bond was then calculated as

$$E_{\text{i}t} = (10E_{f-gra} + 10E_{f-sil} - 20E_{f-gra-sil})/2 \quad \text{(S2)}$$

where $E_{f-gra-sil}$ is the formation energy per atom relative to isolated atoms. For the sake of efficiency, we assume that the interface energy converges for the current five-layer interface model (20 atoms). In principle, we could do a larger model but this would not affect qualitatively the discussions below.

Assuming that the interfaces do not interact each other (actually only valid at very large separation), the interface energy is independent of concentrations. Then the formation energy is linearly proportional to the interface concentration described as follows.

$$E_f = (3n \cdot E_{f-gra} + n \cdot E_{f-sil} - E_i)/(4n) \quad \text{(S3)}$$

$$E_f = (n \cdot E_{f-gra} + 3n \cdot E_{f-sil} - E_i)/(4n) \quad \text{(S4)}$$

where eq. (S3) and eq. (S4) correspond to C/Si= 3/1 in SiC_3 and C/Si= 1/3 in Si_3C, respectively. n is the number of layers of narrower nanoribbons, e.g. Si layers in SiC_3 or C layers in Si_3C, serving as the minimum interface distance. The interface energies E_{i} is defined in eq. (S1) or eq. (S2) depending on the consideration of distortion or not. For the converged and invariant interface energy, the formation energy E_{f} is inversely proportional to the number of separation layers (n).

The results in Figure S3 show that the formation energy of the segregated binary

sheets are always larger than the mixtures if no distortion is considered. However, when the effects of interface-induced distortion are included, the mixture models are more stable than the segregated models with highly concentrated interfaces where the width of interface separation are less than 0.8 and 1.5 layers, respectively, for SiC_3 and Si_3C sheets. The results based on the current models show that the segregated models with highly concentrated interfaces are energetically comparable with the mixture models. Note that the five-layer segregated models may be small for the convergence of interface energy dominated by the distortion. Also, the distortion energy should be larger than the values estimated here in the highly concentrated cases. More accurate quantitative evaluation needs further work, but the qualitative pictures would remain the same. For interfaces at low concentration, the energy penalty arising from the interface formation dominated by the distortion becomes less significant and negligible.

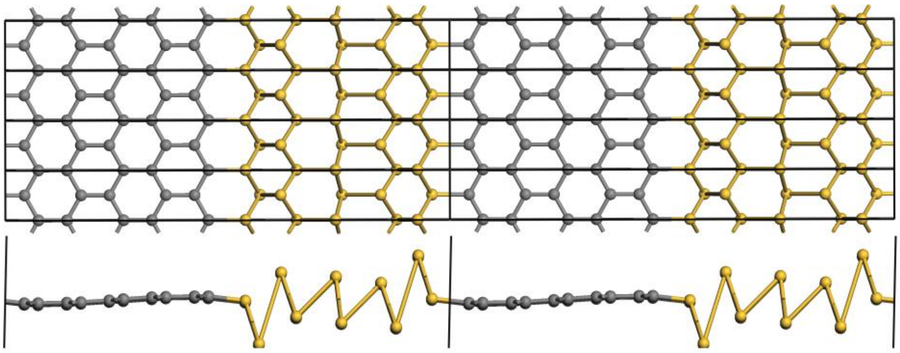

Figure S2 Segregated interface models ($Si_{10}C_{10}$) consisting of five C and Si layers: Top view (top) and side view (bottom).

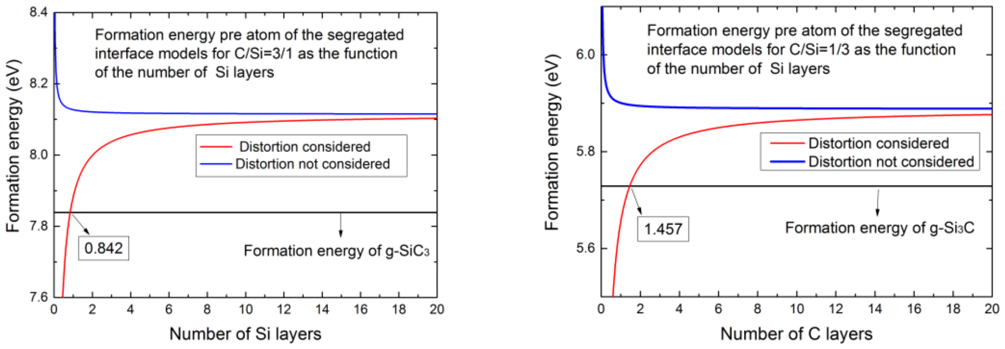

(a) (b)

Figure S3. Formation energies per atom of the segregated interface models for (a) C/Si=3/1 and (b) C/Si=1/3 as the functions of the number of Si and C layers.

S3 Electron transport properties of g-SiC_3 and g-Si_3C nanoribbons

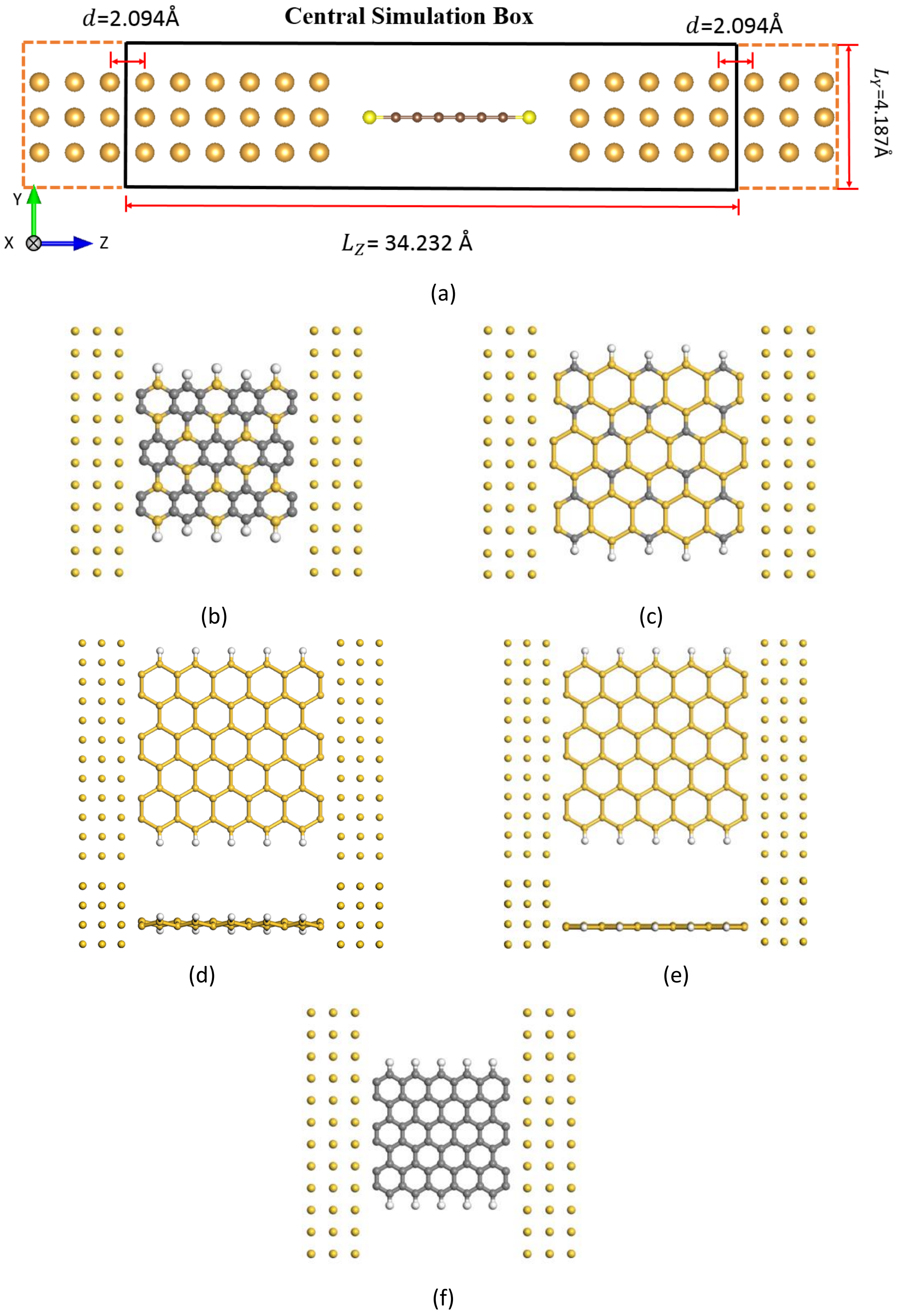

Figure S4. (a) Schematic illustration of central simulation box (side view). Optimized structures of (b) g-SiC_3, (c) g-Si_3C, (d) bulked silicene, (e) planar silicene, and (f) graphene.

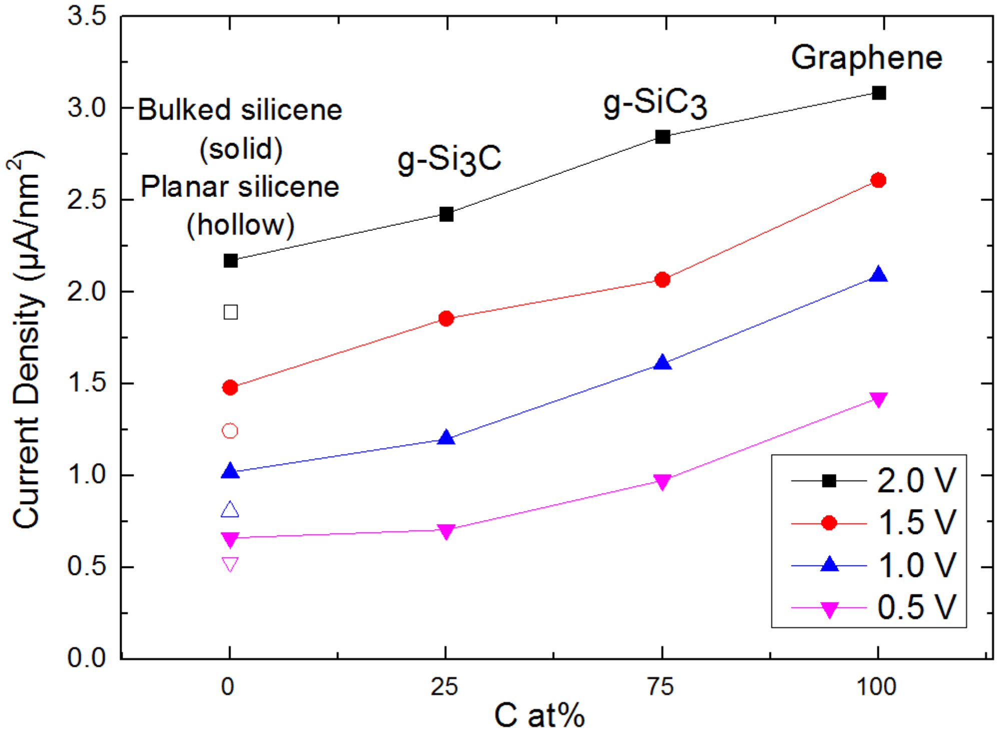

Figure S5. Current vs. carbon concentration curves under bias voltages of 0.5 V (black square), 1.0V (red circle), 1.5V (blue triangle), and 2.0V (pink down triangle).

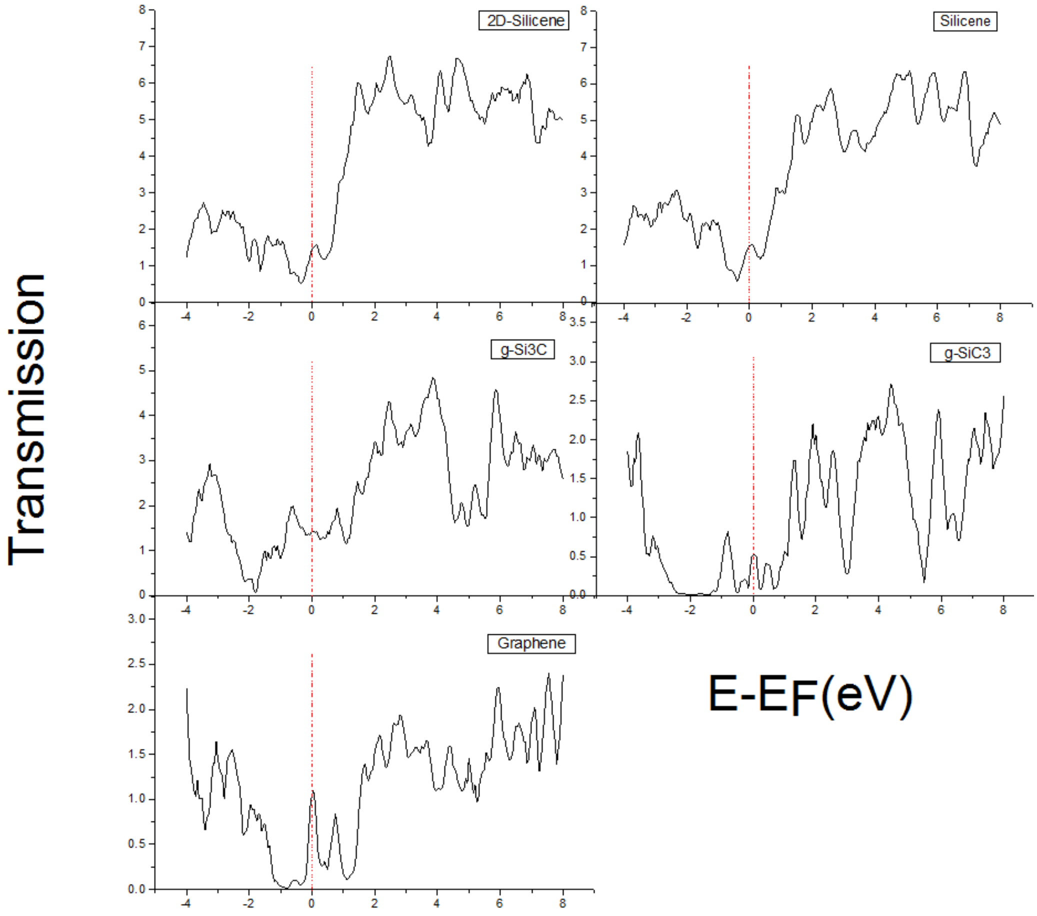

Figure S6. Transmission coefficients of planar silicene, bulkedsilicene, g-Si_3C, g-SiC_3, and graphene from -4.00 to 8.00 in steps of 0.05 (energy unit: eV).

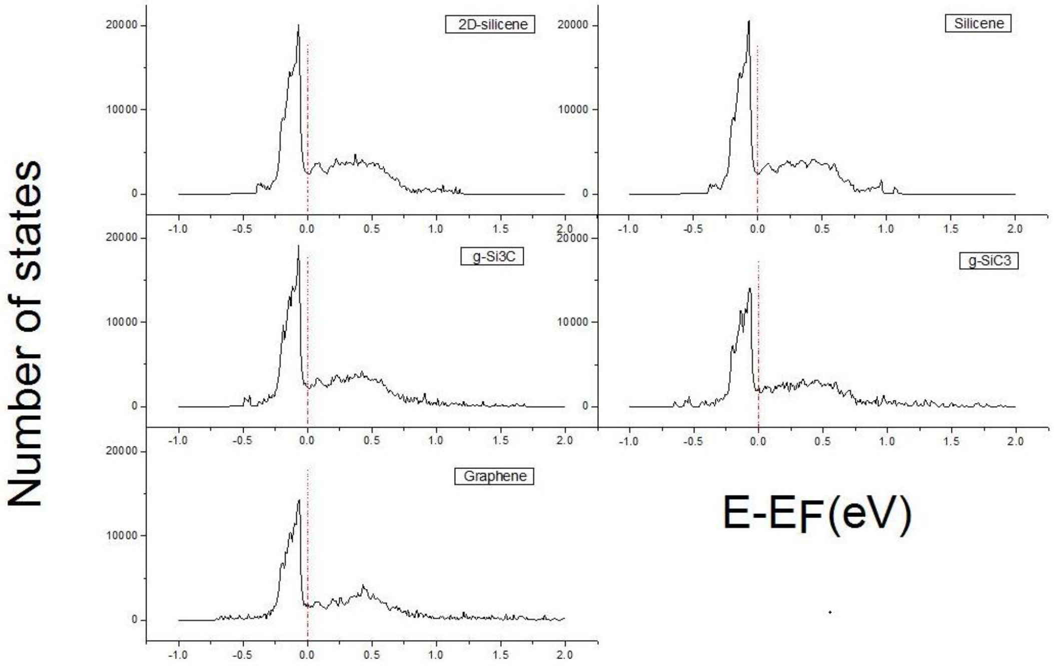

Figure S7. Density of states of planar silicene, bulked silicene, g-Si_3C, g-SiC_3, and graphene from-3.00 to 3.00 in steps of 0.01 (energy unit: eV).

1. Qin, X. M. *et al.* Origin of Dirac Cones in SiC Silagraphene: A Combined Density Functional and Tight-Binding Study. *J. Phys. Chem. Lett.* **6**, 1333-1339 (2015).
2. Qin, X. M., Liu, Y., Chi, B. Q., Zhao, X. L. & Li, X. W. Origins of Dirac cones and parity dependent electronic structures of alpha-graphyne derivatives and silagraphynes. *Nanoscale* **8**, 15223-15232 (2016).
